# Fully automated kidney image biomarker prediction in ultrasound scans using Fast-Unet++

**DOI:** 10.1038/s41598-024-55106-5

**Published:** 2024-02-27

**Authors:** Mostafa Ghelich Oghli, Seyed Morteza Bagheri, Ali Shabanzadeh, Mohammad Zare Mehrjardi, Ardavan Akhavan, Isaac Shiri, Mostafa Taghipour, Zahra Shabanzadeh

**Affiliations:** 1 Research and Development Department, Med Fanavaran Plus Co., Karaj, Iran; 2 Department of Radiology, Hasheminejad Kidney Center, Iran University of Medical Sciences, Tehran, Iran; 3 Section of Body Imaging, Division of Clinical Research, Climax Radiology Education Foundation, Tehran, Iran; 4 Division of Nuclear Medicine and Molecular Imaging, Geneva University Hospital, 1211 Geneva 4, Switzerland

**Keywords:** Biomedical engineering, Computer science

## Abstract

Any kidney dimension and volume variation can be a remarkable indicator of kidney disorders. Precise kidney segmentation in standard planes plays an undeniable role in predicting kidney size and volume. On the other hand, ultrasound is the modality of choice in diagnostic procedures. This paper proposes a convolutional neural network with nested layers, namely Fast-Unet++, promoting the Fast and accurate Unet model. First, the model was trained and evaluated for segmenting sagittal and axial images of the kidney. Then, the predicted masks were used to estimate the kidney image biomarkers, including its volume and dimensions (length, width, thickness, and parenchymal thickness). Finally, the proposed model was tested on a publicly available dataset with various shapes and compared with the related networks. Moreover, the network was evaluated using a set of patients who had undergone ultrasound and computed tomography. The dice metric, Jaccard coefficient, and mean absolute distance were used to evaluate the segmentation step. 0.97, 0.94, and 3.23 mm for the sagittal frame, and 0.95, 0.9, and 3.87 mm for the axial frame were achieved. The kidney dimensions and volume were evaluated using accuracy, the area under the curve, sensitivity, specificity, precision, and F1.

## Introduction

Renal ultrasound plays a critical role in kidney dimension prediction and evaluation of its function. The imaging modality assesses renal anatomy and image guidance for renal interventions^1^. Ultrasound is the modality of choice due to its lower cost, ease of accessibility, lack of radiation, and availability^2^. Several studies indicate the robustness and reliability of volume measurements using ultrasound validated by magnetic resonance imaging (MRI)^3,4^. Specifically, any variations in the anatomical characteristics of the kidney (such as kidney and parenchymal thickness) are associated with clinical disorders. For instance, a small kidney length may indicate subclinical atherosclerotic or irreversible chronic renal disease^4^. In addition, a large kidney length may also be related to higher cardiovascular risks^5^. Moreover, a diminished parenchymal thickness may increase the risk of end-stage renal disease (ESRD) in boys with posterior urethral valves^6^. However, the quality of the ultrasound imaging and its interpretation depend completely on the radiologist’s skills and expertise. On the other hand, investors favor the imaging system since it is a noninvasive diagnostic and screening approach. Many kidney disorders, such as Autosomal dominant polycystic kidney disease (ADPKD), need several follow-ups. Moreover, the kidney length seems to be related to the early assessment of the efficacy of the therapies^7,8^. Despite the significance of determining the kidney’s anatomical characteristics in ultrasound images, the procedure is challenging and suffers from inter and intraobserver variability^4^. Thus, several automatic and semi-automatic approaches have been developed to overcome this issue, including the level-set approach^9^, statistical shape model^10,11^, graph cut^12^, texture analysis^13^, and support vector machine (SVM)^14^. Marsousi et al.^9^ used offline training datasets to generate a 3-D kidney shape model using a proposed shape model representation called the complex-valued implicit shape model. Zheng et al.^12^ drew a graph of image pixels near the boundaries of the kidney instead of creating one of the entire image and combined image pixels with texture feature maps derived from Gabor filters. Xie et al.^13^ utilized ultrasound images’ shape priors combined with their texture. The image texture was obtained by applying a bank of Gabor filters, and the shape priors were gathered using Leventon and Faugeras’s method^15^. Ardon et al.^14^ proposed an SVM method to conquer the high variability in kidney appearance in 3D ultrasound images.

In recent years, several aspects of medical image analysis have been influenced by deep learning^16–22^, and image segmentation has been affected the most. Various deep approaches have been proposed to distinguish the object of interest from its background in medical images. Some include U-net^23^, V-net^24^, SegNet^25^, Fully Convolutional Networks (FCN)^26^, and several promotions of U-net^19,27–30^. These promotions are a testament to the performance and popularity of U-net-based medical image segmentation approaches. Several deep learning-based methods have recently been proposed for kidney image segmentation using boundary distance regression and pixel-wise segmentation^31,32^, 3D U-net^33^, modified FCN^34^, and regular convolutional neural networks (CNN)^35^. Yin et al.^32^ utilized a pre-trained CNN to extract high-level image features from kidney images. They created the boundary distance map of the image using a boundary distance regression network. Finally, the map was fed to a pixel-wise classification network to predict kidney masks. Ravishankar et al. tried to incorporate prior shape information into the FCN through an augmented dataset. They achieved an 83.95% of dice similarity coefficient (DSC) as their best result^34^. In the Supplementary information file, a table is provided comparing kidney segmentation methods using ultrasound images from the literature.

There have been several attempts for kidney segmentation in computed tomography (CT) images. Sharma et al.^35^ concentrated on kidney segmentation in Autosomal Dominant Polycystic Kidney Disease (ADPKD) patients, characterized by kidney enlargement. They utilized a specific architecture of CNN to segment kidneys in CT images. Türk et al. developed a hybrid segmentation model based on V-net^24^ for kidney segmentation on 210 CT images^36^. They furthered their work by utilizing an improved U-Net3D model for kidney segmentation^37^ and a two-stage bottleneck block architecture for renal tumor segmentation^38^ in the same dataset.

The main bottleneck of the proposed methods is the relatively high computational cost of predicting the object mask. This is due to the excessive number of parameters in these models. We have proposed a modification of Fast-Unet to overcome the problem. Fast-Unet is a high-performance novel CNN architecture that aims to segment fetal ultrasound images^39^. The key point in the network is using 2 × 2 stride in the convolution layers that downsamples the spatial resolution of feature maps, thus making the network needless to the pooling layer. Inspired by U-net++, we have introduced Fast-Unet++ architecture, which utilizes diagonal layers to produce the final feature map, thus creating a more precise prediction.

In summary, the main contributions of this manuscript are:

1. We have proposed a novel CNN architecture that accurately segments the kidney in ultrasound images at sagittal and axial views.
2. This is the first contribution introducing a method for automatically predicting all kidney parameters, including its length, width, thickness, volume, and parenchymal thickness.

The latter contribution demonstrates the main clinical value of the proposed method, as the results show. To our knowledge, this is the first attempt to extract five kidney parameters automatically from ultrasound images^31,32,40^. Supriyanto et al.^41^ proposed a semi-automatic approach based on the level set and tried to measure kidney dimensions (including the length, width, thickness, and volume) in only six samples. Kim et al.^42^ developed an automated method for kidney volume measurement in children using ultrasound. However, they only validated the predicted values with the CT dataset.

The proposed network was evaluated for the segmentation and the predicted kidney image biomarkers. In addition, a publicly-available dataset was used to compare the segmentation performance with other related networks^43^. Heretofore, there has been one published article^44^ on segmenting the presented data using nnUnet^45^ and a contrastive learning method proposed by Chaitanya et al.^46^. Therefore, we compared our approach with Fast-Unet, nnUnet, and Chaitanya et al.’s method. Fast-Unet architecture, the promotion implemented to propose Fast-Unet++, the results achieved, and a discussion of the results presented are discussed in the sections below.

## Materials and methods

### Prepared dataset

We trained and validated the proposed architecture for collating kidney ultrasound images in the sagittal and axial views. To ensure sufficient image variability in the training phase, the dataset was collected from several imaging centers with several ultrasound devices from May 2020 to January 2023. The imaging centers include (1) Shahid Hasheminejad, Tehran, Iran; (2) Javadolaemmeh Hospital, Jajarm, Iran; (3) and a private clinic in Bardaskan, Iran. The image acquisition was performed using Luna ultrasound device (SIMUT, Karaj, Iran), Affinity 50 device (Philips, Amsterdam, Netherlands), Voluson 730 Expert ultrasound scanner (General Electric, Austria), and Logiq S7 ultrasound device (General Electric, Austria). In addition, two observers delineated the kidney contours using ImageJ (National Institutes of Health, US). The total dataset consisted of 744 2D ultrasound images in the sagittal and axial views (372 left and 372 right in 372 subjects). The subjects were, on average, 45.2 years old. The male-to-female ratio was 63:37. The dataset was split into 80% for train and 20% for test sets.

The image spacing parameter differs in various datasets, ranging from 0.23 to 0.36 mm/pixel. In addition, the images were captured at different resolutions and resized to 320 × 480 pixels.

Subjects with decreased renal function, abnormal urinalysis findings, renal parenchymal abnormalities, and urinalysis anomalies were excluded. Among the 744 subjects, 13 underwent both kidney ultrasound and CT. The main reason for using CT images was to compare the results of biomarkers’ prediction with the ones achieved from an imaging modality with a higher level of anatomy representation.

### Fast-Unet++

Our previous paper proposed Fast-Unet^39^, which used convolution layers with a 5 × 5 kernel and 2 × 2 stride. Leaky Rectified Linear Units (Leaky ReLU) with 0.2 negative slope coefficient are set as the activation function in the encoder path and the ReLU activation function in the decoder path. The decoder path also used the dropout layer with a probability of 50% and the batch normalization layer.

As described in the paper on Fast-Unet^39^, the architecture incorporates several modifications that enhance its computational efficiency compared to the classical U-Net architecture. These modifications include decreasing the spatial dimension of inputs by convolutional strides rather than max-pooling layers, employing transposed convolutions with stride 2 × 2 for up-sampling, eliminating two additional 2D convolutional layers in the output, and utilizing batch-normalization layers in both encoder and decoder modules. These optimizations (also present in the Fast-Unet++ architecture) collectively reduce the model’s computational complexity significantly compared to classical UNet.

**Figure 1:**
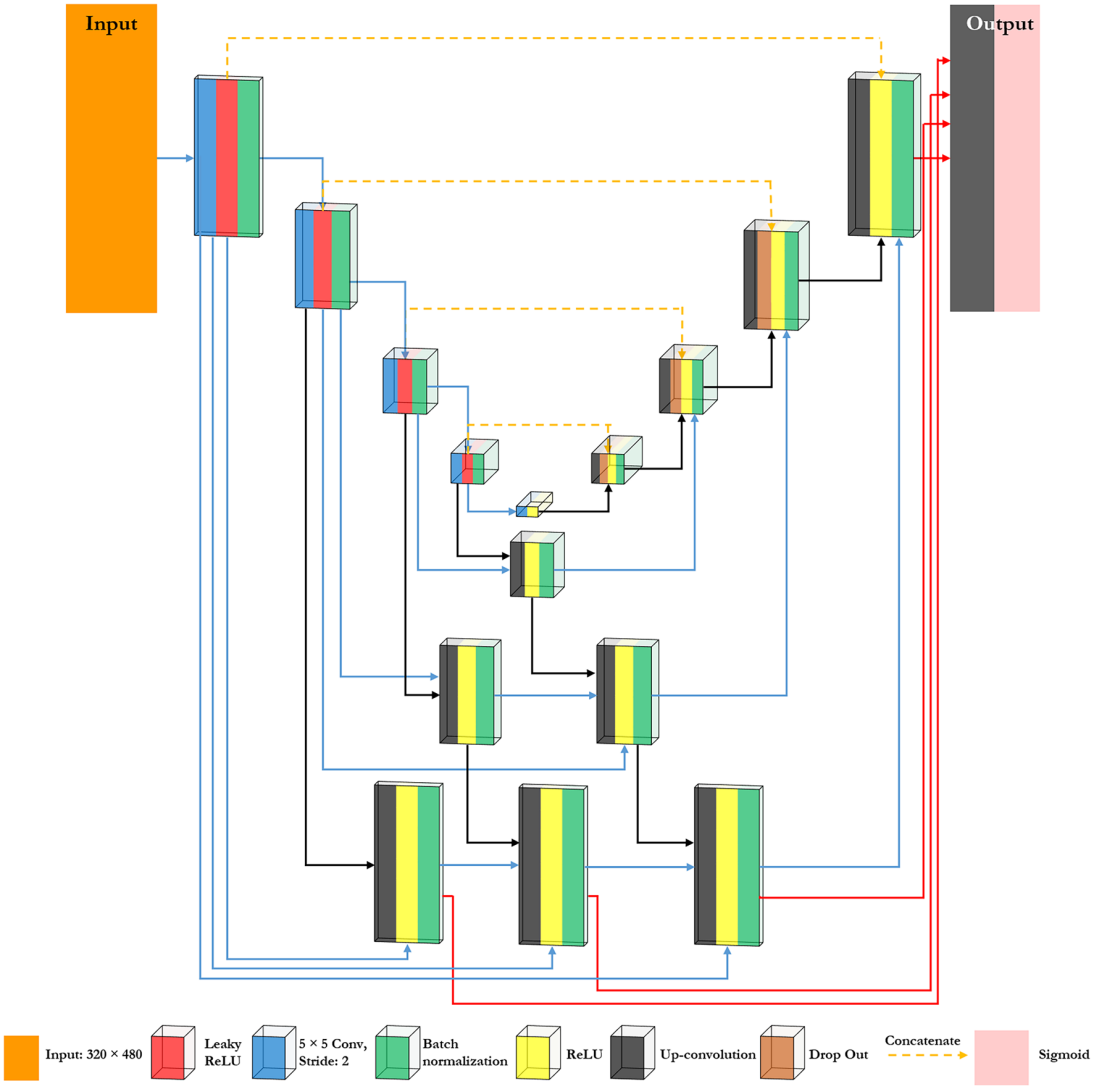
Fast-Unet++ architecture. The nested blocks and deep supervision are the main promotions of Fast-Unet++.

Inspired by UNet+^47^ and MFP-Unet^27^ architectures, we have introduced a nested convolution network in this paper, namely Fast-Unet++. The proposed network has two main advantages over the Fast-Unet architecture, using nested blocks and utilizing all of the feature maps in the top-most level of abstraction for producing the output layer. The latter advantage, known as deep supervision, is presented in our previously published paper, MFP-Unet^27^. Figure 1 depicts the proposed architecture.

As presented in Fig. 1, in the Fast-Unet++ architecture, the skip connection is re-designed. Instead of a direct link between corresponding encoder-decoder blocks, one or more convolution layers are involved in the path. The number of convolution layers and feature maps depends on the level of encoding decoding. These nested convolution blocks are considered as two groups based on their input. For example, group one layers have two inputs consisting of convolution blocks of the same and the next encoding level. On the other hand, group two layers have three inputs comprising (1) a nested layer at the upper level of encoding, (2) a previous nested layer at the same level, and (3) convolution blocks of the same level of encoding. Similar to the last layer of the decoder in the Fast-Unet architecture, the nested layers are composed of up-convolution, ReLU activation function, and batch normalization.

In addition to the transformative nested skip connections and deep supervision, the Fast-Unet++ architecture exhibits several novel features that distinguish it from previous models and contribute to its efficacy in kidney segmentation. First, with feature fusion (three types of inputs listed above in group two), the model can dynamically adjust the importance of feature maps based on local image cues, ensuring that every segmentation decision uses the most relevant data. Moreover, incorporating additional convolution layers in the decoder path enhances feature refinement, leading to smoother and more accurate segmentation boundaries. Moreover, our model implements a progressive refinement strategy in the decoder path, where each nested convolution block refines segmentation details iteratively, culminating in a more granular and contextually informed output. Finally, the model’s robustness to image variations, including noise, artifacts, and variations in kidney morphology, demonstrates its generalizability to a wide range of ultrasound images.

These architectural novelties translate into tangible advancements, delivering not just incremental improvements but a noteworthy leap in performance—evidenced by superior segmentation accuracy in empirical evaluations of both Fast-Unet and UNet++. Our extensive experiments and comparative analyses substantiate the distinctiveness and efficacy of Fast-Unet++ in addressing intricate ultrasound image segmentation challenges.

### Measurement of kidney image biomarkers

**Figure 2:**
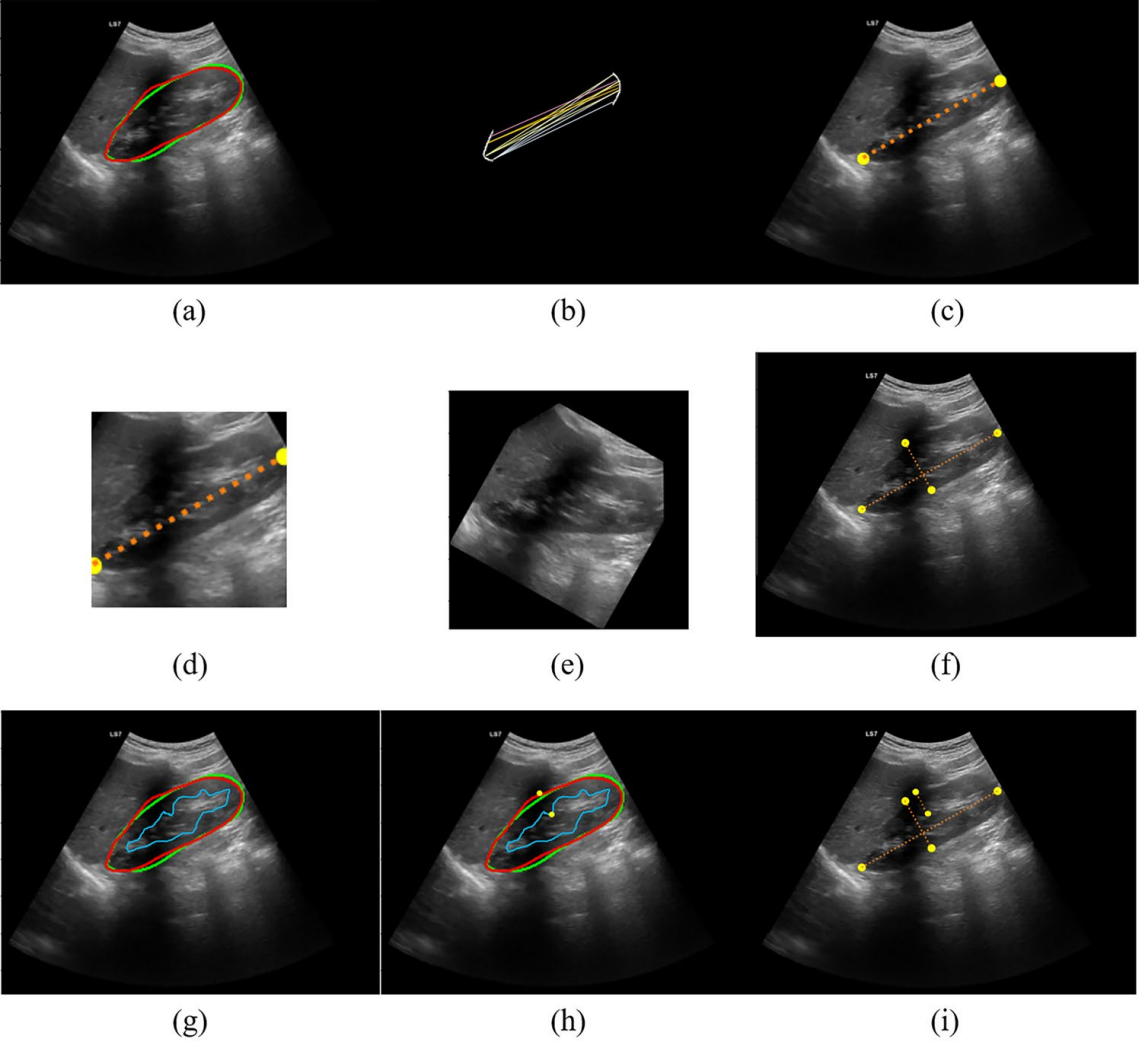
Post-processing of sagittal view image. (**a**) Segmentation of the capsule. The ground truth is the green contour and the predicted mask is the red one. (**b**) Finding the farthest pairs of points between two poles, (**c**) kidney length (KL), (**d**) tailored crop, (**e**) rotation of the image to make it horizontal, (**f**) finding kidney thickness, (**g**) segmentation of sinus, (**h**) finding the farthest pairs of points between sinus and capsule’s masks, and (**i**) parenchyma length.

After kidney segmentation in sagittal and axial views, post-processing algorithms were performed to calculate the renal dimensions and volume. These algorithms, grouped based on the input image and illustrated in Fig. 2, are presented in the following:

#### Post-processing of sagittal images

The segmented area of the sagittal-view images was used to calculate the kidney length (KL), thickness (KT), and parenchymal thickness. Figure 2a–e represents the post-processing related to the calculation of KL. Figure 2a shows the segmented kidney capsule in the sagittal view image. The green contour represents the ground truth, delineated by the expert radiologist, and the red contour represents the predicted contour by Fast-Unet++. The upper and lower poles of the kidney, which were extracted from the mask, are shown in Fig. 2b. The farthest pairs of points between the two poles were found using a grid search algorithm. The colored lines in Fig. 2b illustrate the procedure, and the determined points are shown in Fig. 2c. The KL is the distance between these two points.

The second row of Fig. 2 is related to the calculation of the kidney thickness. First, the image was cropped using a tailored-crop algorithm based on two points annotating the KL^48^. The resulting square-shaped image enclosed the kidney and some of the background, as shown in Fig. 2d. Next, the cropped image was rotated-padded based on the angle achieved from the KL’s line. In this way, the kidney would be horizontal (Fig. 2e). Finally, to achieve the kidney thickness, the topmost point of the rotated mask was found, and the corresponding downmost point was reached. These points annotate the kidney thickness depicted in Fig. 2f.

The algorithm of the parenchymal thickness calculation is shown in the third row of Fig. 2. The kidney sinus was segmented using Fast-Unet++, and the farthest points between the upper contour of the kidney mask and the sinus mask were found (Fig. 2g, h). The distance between these two points is considered the parenchymal thickness.

#### Post-processing of axial images

**Figure 3:**
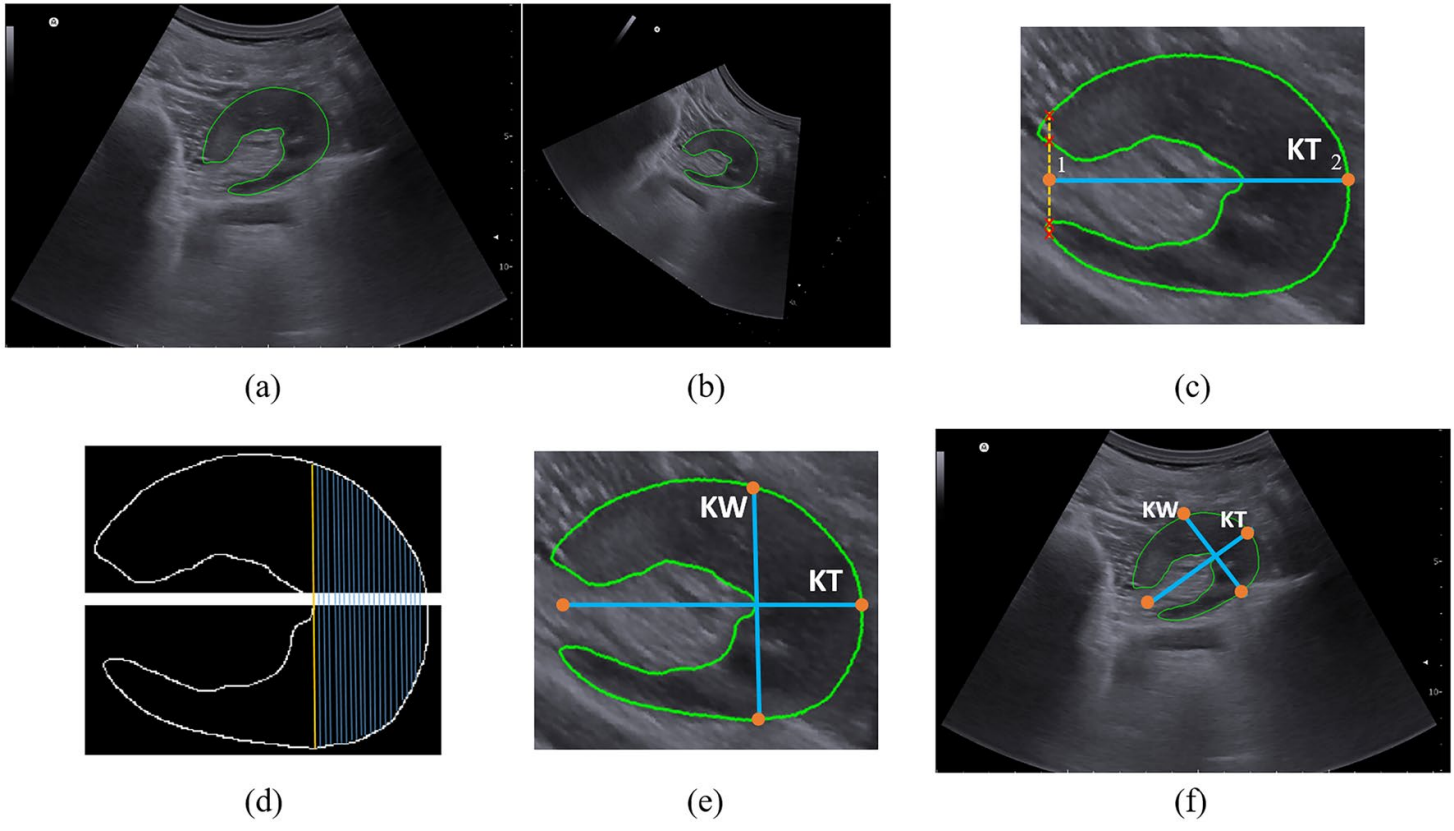
Post-processing of axial view image. (**a**) Segmentation of axial image, (**b**) rotation of the image to make it horizontal, (**c**) estimating kidney thickness (KT) by finding two terminal points, (**d**) separation of upper and lower parts of the mask and finding the longest distance, (**e**) kidney width (KW) and KT, and (**f**) KW and KT in the original image.

We applied an algorithm to calculate kidney thickness using the axial plane, which some radiologists prefer. Figure 3 shows that the kidney thickness was measured in the axial plane as the maximum length parallel to the hilum. After the kidney segmentation, the major axis of the mask was calculated using the first eigenvector of the principal component analysis (PCA). Then, the mask was rotated horizontally using the angle achieved from eigenvectors (Fig. 3b). When the level of the hilum is on the left, the rightmost point of the mask represents the kidney border terminal and vice versa. This point is shown as point 2 in Fig. 3c. Point 1 was determined by finding the first line that passes through four points in the mask contour and assigning the middle point as the point.

The upper and lower parts of the contour were separated to measure the kidney width. For each point in the upper part, the nearest point in the lower part was found. Therefore, the longest distance between these pairs of points was determined as the kidney thickness (Fig. 3d, e). Finally, the rotation algorithm is applied in the opposite direction to achieve the real places of the determined points, as shown in Fig. 3f.

#### Kidney volume prediction

According to various publications^3,4,49^, the kidney volume extracted from ultrasound images highly correlates with the values extracted with MRI and CT scans. KL and KT are needed for predicting kidney volume, measured from the sagittal view image, and KW from the axial view image. Thus, the kidney volume was calculated using the following formula:

1 $$Kidney\;Volume = KL \times KW \times KT \times 0.523$$

### Implementation details and evaluation metrics

The proposed model was implemented using Python 3.8.12, Tensorflow 2.3.0, and Keras 2.4.3. The batch size was 32, and the maximum number of iteration steps was 47. The hardware used to train the deep learning model contained a GeForce RTX 2060 Graphics processing unit (GPU), HP 32GB DDR4 RAM, and Intel Core i5-7400 CPU. The Adam stochastic optimization^50^ was used to compile the model with a learning rate of 10e-4. A random normal initializer with a mean of 0 and a standard deviation of 0.02 was used to initialize the filters of the proposed model. The hyperparameter optimization method used to select the best parameters (including the learning rate, the batch size, and the convolution kernel size) was a grid search. The grid search consisted of training the model with a range of values for each parameter and selecting the ones producing the best results. The results were evaluated using the mean dice similarity coefficient (DSC)^51^.

Segmentation performance was measured using DSC, Jaccard coefficient (JC)^52^, and mean absolute distance (MAD)^53^. The predicted measurements were also evaluated using accuracy, the area under the curve (AUC), sensitivity, specificity, precision, and F1 score^54,55^.

The convolutional layers dominate the computational complexity of the proposed model. The number of floating-point operations (FLOPs) required to compute the output of a convolutional layer is given by:

2 $$FLOPs = 2K2 \times C_{in} \times C_{out} \times N$$

where K is the kernel size of the convolutional filter. C_in_ is the number of input channels. C_out_ is the number of output channels. N is the number of pixels in the input image.

For the Fast-Unet++ model, the total number of FLOPs is approximately 1.5e10.

## Result

**Figure 4:**
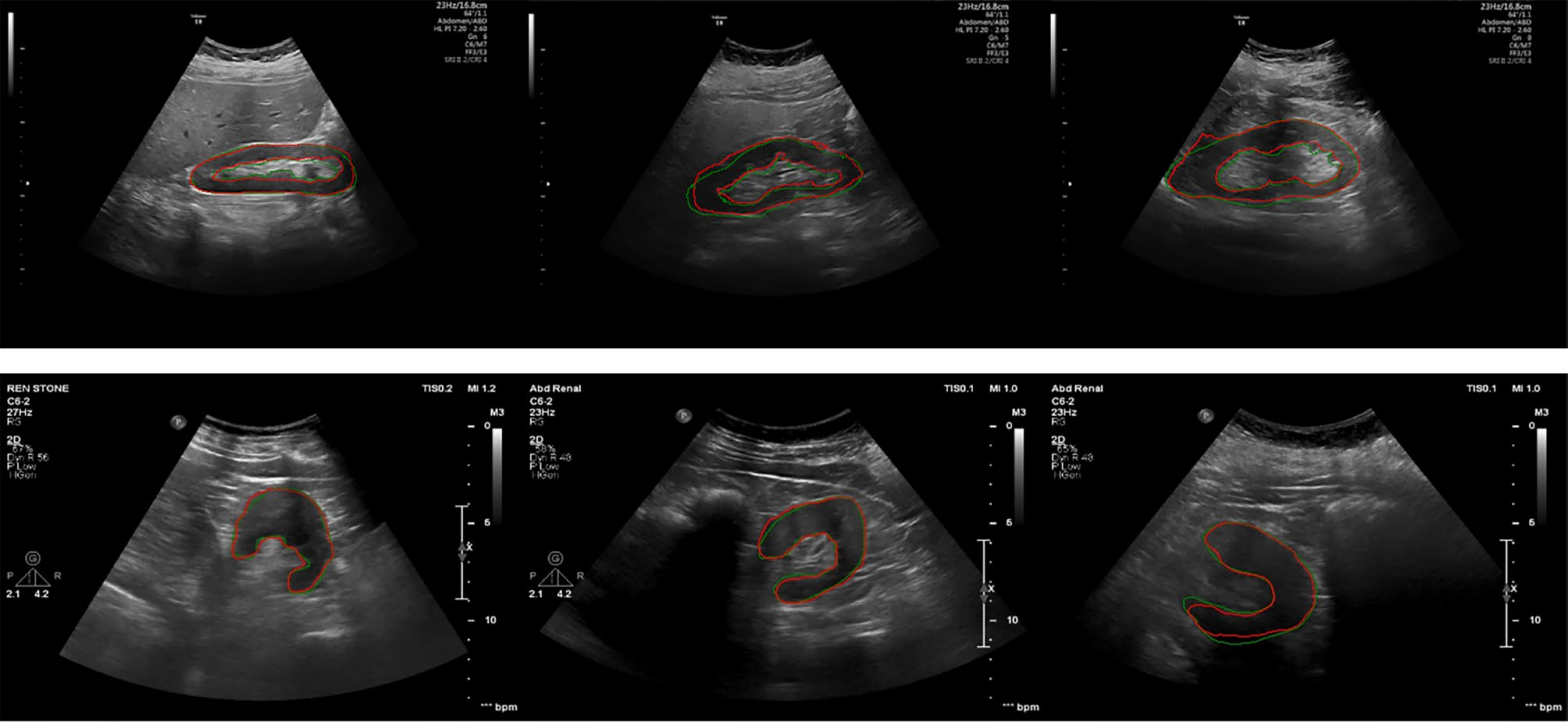
Segmentation results of the ultrasound kidney in the sagittal (top row) and axial (bottom row) images. The green contours represent the manual segmentation and the red contours represent automatic results.

Figure 4 shows the results of the segmentation network on ultrasound images of the kidney in sagittal and axial views. In this Figure, the green contour shows the ground truth that an expert delineates, and the red contour depicts the predicted mask by the proposed network. The promising agreement between the manual and automatic contour proves the robustness of the proposed method.

**Figure 5:**
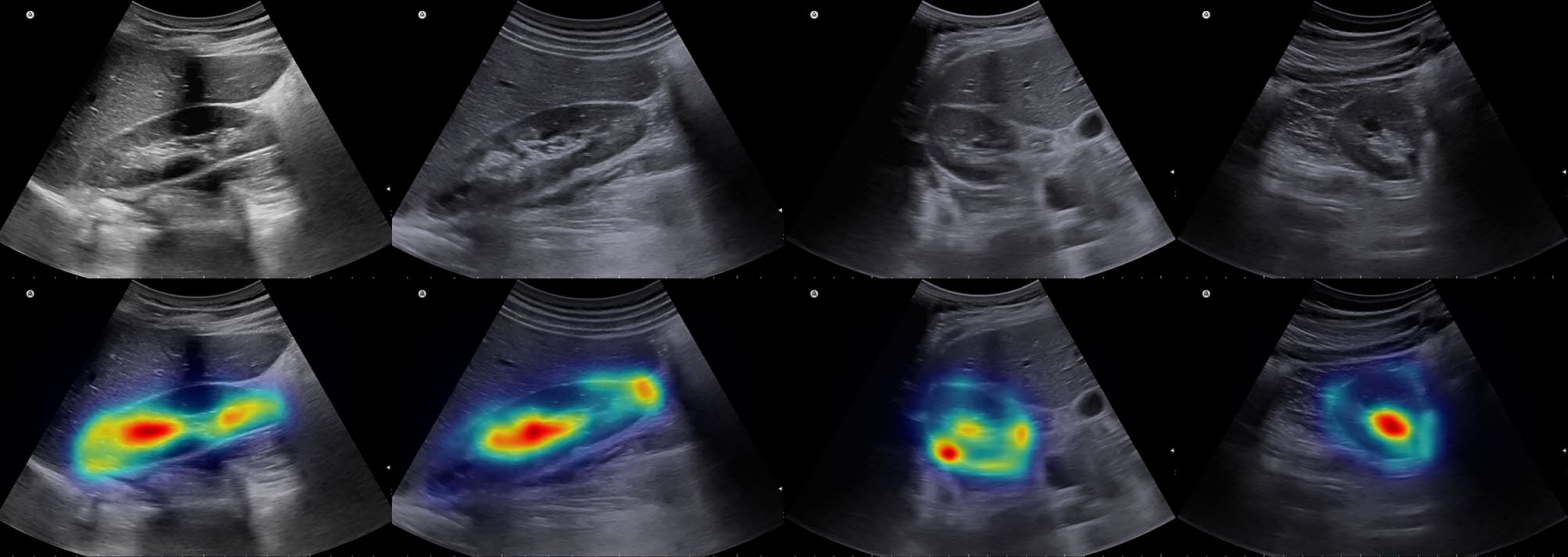
Grad-CAM analysis results in some samples of sagittal and axial images. Top row is the original images and bottom row is the resulted grad-CAM analysis.

To enhance the interpretability of the proposed Fast-Unet++ architecture, we applied grad-CAM analysis to visualize the model’s activation patterns and identify the regions that contribute most significantly to the segmentation predictions. The results are visualized in Fig. 5.

We also calculated DSC for 100 random samples of the test image set and presented them in a Swarm scatter plot, a scatter plot with the points offset (jittered) in the x-dimension. In this Figure (introduced in the Supplementary information file), sagittal images are labeled as one, and axial images are labeled as two. As the Figure shows, the network performs better in s sagittal than axial images.

Speckle noise is a common artifact in ultrasound images that can degrade the performance of image segmentation models. Previous studies have shown that denoising algorithms can improve the segmentation accuracy of ultrasound images^56,57^. We evaluated the performance of our Fast-Unet++ model on ultrasound images with and without denoising. We used the adaptive bilateral filter^58^ method for denoising. We found that denoising did not significantly improve the segmentation accuracy of our model.

We believe that our Fast-Unet++ model is designed to be robust to noise. The model uses skip connections to allow information from earlier layers of the network to be propagated to later layers. This helps the model to learn to distinguish between relevant features and noise.

Quantitative analysis of the proposed segmentation network using our prepared dataset was performed using DSC, JC, and MAD metrics and compared with the results of two other related networks (Fast-Unet and UNet + +) to evaluate how our proposed modifications influence the results. Table 1 shows the results based on imaging views (sagittal and axial). The achieved metrics in sagittal view images are better than those achieved in axial view images, which was expected according to our previously presented results.

**Table 1:** Evaluation of the segmentation of kidney images. The values are with the form of (mean ± standard deviation).

Network	View	DSC	JC	MAD
Fast-Unet++	Sagittal	0.97 ± 0.02	0.94 ± 0.01	3.23 ± 0.89
	Axial	0.95 ± 0.04	0.90 ± 0.00	3.87 ± 1.02
Fast-Unet	Sagittal	0.94 ± 0.01	0.91 ± 0.01	4.73 ± 0.93
	Axial	0.83 ± 0.0	0.8 ± 0.02	6.83 ± 1.23
UNet++	Sagittal	0.91 ± 0.02	0.85 ± 0.02	4.99 ± 1.1
	Axial	0.84 ± 0.0	0.79 ± 0.05	6.07 ± 012

*DSC* Dice similarity coefficient, *JC* Jaccard coefficient, *MAD* Mean absolute distance.

Beyond that, the network was compared with several networks using a publicly-available dataset^43^. The compared models contained Fast-Unet^39^, nnUnet^45^, SwinUNETR^59^, UNet++^47^, DeepLabV3 + ^60^, FCN^26^, PSPNet^61^, traditional U-Net^23^, and a method proposed by Chaitanya et al.^46^. The dataset was collected in 5 years from over 500 patients. All images were categorized into three groups according to the acquisition view. The ground truth annotation was delineated for the kidney capsule, cortex, medulla, and sinus. Since our proposed method was trained for segmenting the kidney capsule and sinus, we compared our results in these two regions. Some of the results presented by Singla et al.^44^ and the rest presented by Valente et al.^62^, used different evaluation metrics; therefore, we quoted the results from both studies with their own metrics. The results are presented in Table 2. Due to the dataset in this study providing segmentation from two experts, Table 2 shows the predictions based on both ground truths. As Singla et al.^44^ did not report their results based on two experts’ masks, just one could be presented.

**Table 2:** Evaluation of the segmentation of kidney images using the public dataset. The HD^1^ and SSD^2^ metrics are reported on millimeters.

Network	Capsule/s1	Sinus/s1	Capsule/s2	Sinus/s2
Proposed method	DSC = 0.95 HD = 2.03 SSD = 2.9	DSC = 0.87 HD = 4.7 SSD = 3.2	DSC = 0.96 HD = 2 SSD = 2.45	DSC = 0.88 HD = 4.12 SSD = 2.98
Fast-Unet	DSC = 0.93 HD = 4.15 SSD = 4.3	DSC = 0.87 HD = 4.73 SSD = 5.5	DSC = 0.93 HD = 2.78 SSD = 3.81	DSC = 0.88 HD = 4.11 SSD = 4.2
nnUnet	DSC = 0.86 HD = 10.8	DSC = 0.77 HD = 8.6	–	–
Chaitanya et al	DSC = 0.82 HD = 10.9	DSC = 0.7 HD = 9	–	–
DeepLabV3 +	DSC = 0.93 SSD = 4.3	DSC = 0.82 SSD = 4.7	DSC = 0.94 SSD = 3.8	DSC = 0.83 SSD = 4.2
SwinUNETR	DSC = 0.92 SSD = 5.4	DSC = 0.81 SSD = 5.4	DSC = 0.92 SSD = 6.2	DSC = 0.84 SSD = 5.2
UNet++	DSC = 0.92 SSD = 6.3	DSC = 0.83 SSD = 5.0	DSC = 0.92 SSD = 6.6	DSC = 0.85 SSD = 4.3
FCN	DSC = 0.93 SSD = 4.6	DSC = 0.8 SSD = 5.1	DSC = 0.94 SSD = 4	DSC = 0.8 SSD = 4
PSPNet	DSC = 0.92 SSD = 5.6	DSC = 0.81 SSD = 5.6	DSC = 0.92 SSD = 5.8	DSC = 0.84 SSD = 5.4
U-Net	DSC = 0.94 SSD = 3.9	DSC = 0.84 SSD = 4.0	DSC = 0.95 SSD = 3.6	DSC = 0.88 SSD = 3.6

*DSC* Dice similarity coefficient, *HD* Hausdorff distance, *SSD* Symmetric surface distance.

The final evaluation of the proposed network compares the predicted kidney mage biomarkers (length, width, thickness, volume, and parenchymal thickness) with those achieved via CT scan. As mentioned, 13 cases had CT images, along with kidney ultrasounds. We used accuracy, AUC, sensitivity, specificity, precision, and F1 score to compare the results. According to Table 3, the proposed method produces robust results across all metrics. To be more accurate, we used the kidney width estimated from sagittal images in the present comparison.

**Table 3:** Comparing the predicted kidney dimension in ultrasound images with the results of CT images. The values are in the form of (mean ± standard deviation).

Metric	Dimension				
	Length	Width	Thickness	Parenchyma	Volume
Accuracy	0.96 ± 0.01 p-value < 1e-5	0.95 ± 0.01 p-value < 1e-5	0.92 ± 0.00 p-value < 1e-5	0.89 ± 0.03 p-value = 7e-5	0.91 ± 0.03 p-value = 2e-4
AUC	0.95 ± 0.01 p-value = 1e-4	0.94 ± 0.01 p-value = 2e-4	0.89 ± 0.03 p-value < 1e-5	0.89 ± 0.04 p-value = 5e-4	0.91 ± 0.00 p-value = 6e-5
Sensitivity	0.89 ± 0.00 p-value < 1e-4	0.91 ± 0.00 p-value = 3e-4	0.88 ± 0.00 p-value < 1e-5	0.88 ± 0.01 p-value = 2e-4	0.9 ± 0.04 p-value = 5e-4
Specificity	0.91 ± 0.02 p-value < 1e-5	0.90 ± 0.02 p-value < 1e-5	0.89 ± 0.01 p-value = 7e-5	0.83 ± 0.00 p-value = 6e-5	0.9 ± 0.02 p-value < 1e-5
Precision	0.95 ± 0.02 p-value < 1e-5	0.95 ± 0.02 p-value < 1e-5	0.9 ± 0.02 p-value = 8e-4	0.87 ± 0.01 p-value = 7e-5	0.89 ± 0.03 p-value < 1e-5
F1	0.92 ± 0.02 p-value = 1e-3	0.93 ± 0.02 p-value < 1e-5	0.89 ± 0.02 p-value = 7e-4	0.87 ± 0.03 p-value < 1e-5	0.9 ± 0.02 p-value < 1e-5

*AUC* Area under the curve.

## Discussion

In this study, we proposed a novel CNN-based model for kidney segmentation from ultrasound images in sagittal and axial views and predicting kidney image biomarkers and volume. As far as we know, this is the first attempt to predict three kidney dimensions in addition to its volume and parenchymal thickness. We developed our previously published model, Fast-Unet^39^, by adding nested layers inspired by Unet++^47^. Compared with these networks, Fast-Unet++ takes advantage of the low computation cost of Fast-Unet and nested layers of Unet++. Therefore, combining these two structures yields better results, as reported in Table 1. Fast-Unet, however, performed better than Unet++, explainable by the intrinsic features of its architecture. As quantitative and qualitative results show, segmentation of the kidney in the sagittal frame yields more satisfying results due to the clearer borders of the kidney in these images. Thus, this project did not use an axial view for parenchymal thickness, as some radiologists do. In addition, the grad-CAM results demonstrate that the model effectively focuses on the kidney region, with high activation values highlighting the key anatomical features that guide the segmentation process. This analysis provides valuable insights into the model’s decision-making process and reinforces its ability to accurately segment the kidney in ultrasound images.

A comprehensive dataset was acquired from various imaging centers and imaging vendors (GE, Philips, and Simut) for the training and evaluation of the network. Since the ultrasound images of the kidney are affected by the operator’s experience, the imaging system, and the defined preset, collecting such an extensive dataset provides an illustrative vision of the network’s performance.

We compared the network’s performance with the CT images as the gold standard since the final goal of this study was to predict clinically routinely measured dimensions of the kidney. According to the results, our proposed model reliably represents all kidney dimensions, especially in length and width. This was predictable because sagittal images had higher quality, and kidney borders were more clearly defined than sinus borders. In addition, the evaluation of the kidney volume shows acceptable values in all metrics. To our knowledge, Kim et al.′s^42^ study is the only paper comparing kidney volumes predicted using artificial intelligence from ultrasound images with CT images. Their results show a 90% correlation between the predicted and reference values. While they have shown promising results, their work suffers from a low number of training images and evaluation metrics. Moreover, they have used 3D ultrasound, which provides more accurate images.

Another evaluation procedure of the proposed method was comparing the results with two other CNNs using a public dataset^43^. Since nnUnet^45^ and Chaitanya et al.’s approach^46^ have been tested on this public dataset, comparing the results of our proposed model with them brings a good insight into the model’s performance. As results show, the proposed model achieved better results in terms of DSC and HD. Although the MAD metric was calculated, it was not comparable because the authors did not provide this information in their paper. The main advantage of the proposed model that causes this superiority is that it is less complicated than those two.

One of the most important limitations of this work was that the radiologist’s skill in acquiring the input image directly influences the result of segmentation. Some models have been introduced to overcome this issue. For instance, van den Heuvel et al.^63^ proposed a model to automatically detect the fetal head and then estimate head circumference (HC) from a sequence of 2D ultrasound frames obtained using the obstetric sweep protocol (OSP). Their paper resulted from a program aimed at helping expand ultrasound in developing countries, which suffer from a lack of trained sonographers, with the help of artificial intelligence. This approach can be implemented for kidney image biomarker prediction, too. In other words, the sonographer sweeps the ultrasound transducer over the patient’s skin using a predefined protocol, which completely contains sagittal and axial views of the kidney. Then, a CNN model analyzes the frames to detect the best sagittal and axial ones. Finally, the image segmentation would be applied to the best frames.

The other limitation is the definition of the best frame for the estimation of kidney length and parenchymal thickness. According to the definition, kidney length is estimated in a frame where the largest view of the kidney is achieved. At the same time, this is not necessarily an excellent option for predicting parenchymal thickness since it needs a proper contrast between the sinus and parenchyma.

In addition, some abnormal structures, such as polycystic kidney (PKD) or tumors, can alter the kidney’s appearance and make it more difficult for the model to identify the correct segmentation boundaries^64^. PKD is a genetic disorder characterized by the development of numerous fluid-filled cysts within the kidneys. These cysts can vary in size and distribution, dramatically altering the kidney’s morphology and complicating the segmentation process. Tumors, on the other hand, can have a variety of appearances, ranging from solid masses to cystic lesions. Their irregular shapes and heterogeneous echogenicity can make it difficult for the model to distinguish them from normal kidney tissue. To address these challenges, future studies may focus on utilizing prior knowledge about the characteristics of abnormal structures to guide the model’s segmentation and employing ensemble methods by combining multiple models, each trained on different aspects of the image, such as texture, shape, and intensity.

This work can be extended by predicting the thickness of the renal medulla and cortex, which are great representatives of kidney functions and can show disorders such as chronic kidney disease (CKD)^65^. This is achievable by a high-quality dataset in which the medulla appears clearly in sagittal images. However, this raises an important issue: differentiating between medulla and kidney cysts because the medulla appears hypoechoic in ultrasound images.

## Conclusion

The proposed CNN architecture demonstrated robust kidney segmentation in both sagittal and axial frames. The model achieved promising values for kidney image biomarkers (including length, width, thickness, and parenchymal thickness) and kidney volume, particularly for sagittal frames. These results suggest that the model can be applied to clinical settings for kidney assessment. Future work should focus on improving the accuracy of parenchyma volume estimation and exploring the model’s performance in a larger and more diverse dataset.

### Supplementary Information

Supplementary Information.

## Data Availability

The public kidney dataset (Open Kidney Dataset) is available on https://rsingla.ca/kidneyUS/. Our prepared dataset is available from the corresponding author upon reasonable request.
