# Supplementary material for: Fully automated kidney image biomarker prediction in ultrasound scans using Fast-Unet++
Source: Sci Rep. 2024 Feb 27;14:4782. doi: 10.1038/s41598-024-55106-5 (PMC10899245; doi:10.1038/s41598-024-55106-5)
Supplement: Supplementary file 1 — Supplementary Information. [file 41598_2024_55106_MOESM1_ESM.docx]

Table S1. A comparative analysis of kidney segmentation methods in ultrasound images

| Method | Method | Dataset | Evaluation Metrics | Limitations |
| --- | --- | --- | --- | --- |
| Proposed method | CNN: Fast-Unet++ | 744 sagittal and axial images | DSC, JC, MAD | Polycystic morphology |
| Chen et al. (2023) ^S1^ | CNN: Asymmetric U-shaped network with hybrid attention mechanism | 300 sagittal images | Accuracy, DSC, JC, Recall, Precision, and ASSD | 1. False detections and missed detections due to the inﬂuence of image quality, blurred boundaries and heterogeneous structure 2. Not considering the disturbance of speckle noise to segmentation results 3. Not considering the real-time nature of ultrasound |
| Peng et al. (2023) ^S2^ | CNN: Deep fusion learning network + searching polygon tracking | 380 sagittal images obtained from 115 patients | DSC, JC | Memory burden of the method due to searching polygon tracking method |
| Song et al. (2023) ^S3^ | CNN: Shape aware dual-task multi-scale fusion network + a self-correction strategy | - | DSC, JC, HD, and ASSD | Geometric shape constraints |
| Alex et al. (2022) ^S4^ | CNN: YSegNet (based on VGG-16) | 700 sagittal images | Accuracy, DSC, JC, Speciﬁcity, Recall, and Precision | VGG-16 is a heavy network that has a lot of parameters to learn. The authors tried to compensate with augmentation |
| Chen et al (2022) ^S5^ | CNN | 400 sagittal images | Accuracy, DSC, JC, Recall, Precision, and ASSD | 1. False detections and missed detections due to the inﬂuence of image quality, blurred boundaries and heterogeneous structure 2. Not considering the real-time nature of ultrasound |
| Chen et al (2021) ^S6^ | CNN: SDFNet | 500 sagittal images | Accuracy, IoU, Precision, Recall, Specificity, and F1 | 1. Missed detection and false detection on individual images. 2. Not considering the real-time nature of ultrasound |
| Yin et al (2020) ^32^ | CNN + boundary distance regression | 289 sagittal images | DSC, MD, JC, Recall, Precision, and ASSD | Dependency of the method to the shape of training data, decreases the performance of method for axial images. |
| Ravishankar et al. (2017) ^34^ | CNN: FCN | 231 sagittal images | DSC | Large number of training parameters of FCN that needs more training images. |
| Ardon et al (2015) ^14^ | SVM + model based deformation | 480 3D ultrasound images | DSC | Dependency of the final result to the SVM |

**CNN:** Convolutional neural network, **DSC:** Dice similarity coefficient, **JC:** Jaccard coefficient, **MAD:** Mean absolute distance, **ASSD:** Average symmetric surface distance, **HD:** Hausdorff distance, **IoU:** Intersection over Union, **FCN:** Fully convolutional network


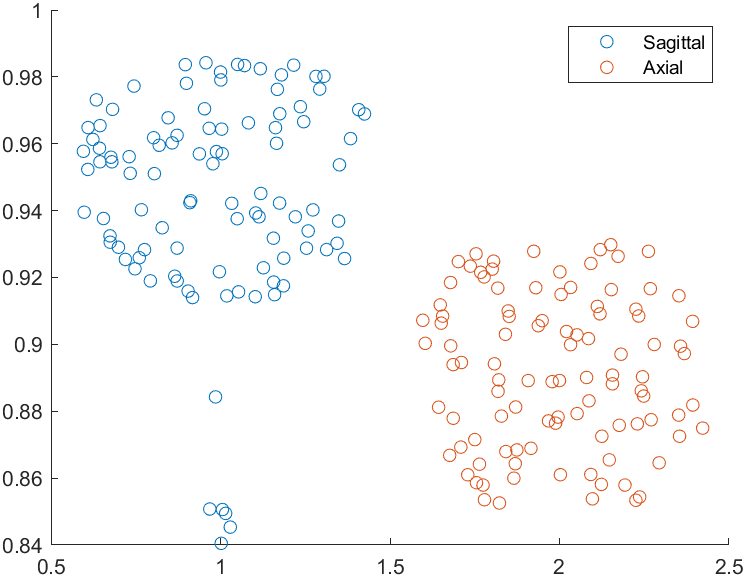


Figure S1. Swarm scatter plot of the DSC achieved on the 100 samples of sagittal and axial images in the test set.

**References**

1. Chen, G. P. *et al.* Asymmetric U-shaped network with hybrid attention mechanism for kidney ultrasound images segmentation. *Expert Syst. Appl.* **212**, 118847 (2023).
2. Peng, T., Gu, Y., Ruan, S. J., Wu, Q. J. & Cai, J. Novel Solution for Using Neural Networks for Kidney Boundary Extraction in 2D Ultrasound Data. Biomol. 2023, Vol. 13, Page 1548 13, 1548 (2023).
3. Song, Z., Liu, X., Gong, Y., Hao, T. & Zeng, K. A Two-Stage Framework for Kidney Segmentation in Ultrasound Images. 60–74 (2023) doi:10.1007/978-981-99-5847-4_5.
4. Alex, D. M., Abraham Chandy, D., Hepzibah Christinal, A., Singh, A. & Pushkaran, M. YSegNet: a novel deep learning network for kidney segmentation in 2D ultrasound images. Neural Comput. Appl. 34, 22405–22416 (2022).
5. Chen, G. et al. A novel convolutional neural network for kidney ultrasound images segmentation. Comput. Methods Programs Biomed. 218, 106712 (2022).
6. Chen, G. *et al.* SDFNet: Automatic segmentation of kidney ultrasound images using multi-scale low-level structural feature. *Expert Syst. Appl.* **185**, 115619 (2021).
